# Microclimatic Divergence in a Mediterranean Canyon Affects Richness, Composition, and Body Size in Saproxylic Beetle Assemblages

**DOI:** 10.1371/journal.pone.0129323

**Published:** 2015-06-05

**Authors:** Jörn Buse, Samuel Fassbender, Martin H. Entling, Tomas Pavlicek

**Affiliations:** 1 University Koblenz-Landau, Institute for Environmental Sciences, Ecosystem Analysis, Fortstrasse 7, Landau, Germany; 2 University of Haifa, Institute of Evolution, Mt. Carmel, Haifa, Israel; National Cancer Institute, UNITED STATES

## Abstract

Large valleys with opposing slopes may act as a model system with which the effects of strong climatic gradients on biodiversity can be evaluated. The advantage of such comparisons is that the impact of a change of climate can be studied on the same species pool without the need to consider regional differences. The aim of this study was to compare the assemblage of saproxylic beetles on such opposing slopes at Lower Nahal Oren, Mt. Carmel, Israel (also known as “Evolution Canyon”) with a 200–800% higher solar radiation on the south-facing (SFS) compared to the north-facing slope (NFS). We tested specific hypotheses of species richness patterns, assemblage structure, and body size resulting from interslope differences in microclimate. Fifteen flight-interception traps per slope were distributed over three elevation levels ranging from 50 to 100 m a.s.l. Richness of saproxylic beetles was on average 34% higher on the SFS compared with the NFS, with no detected influence of elevation levels. Both assemblage structure and average body size were determined by slope aspect, with more small-bodied beetles found on the SFS. Both the increase in species richness and the higher prevalence of small species on the SFS reflect ecological rules present on larger spatial grain (species-energy hypothesis and community body size shift hypothesis), and both can be explained by the metabolic theory of ecology. This is encouraging for the complementary use of micro- and macroclimatic gradients to study impacts of climate warming on biodiversity.

## Introduction

Climate change is considered to be one of the main drivers directly affecting vegetation composition, ecosystem functioning and biodiversity in forests [1,2,3,4,5,6,7]. Forests are facing global increases in frequency and duration of drought events and heat stress as well as frequency of wild fires and windthrows. This process is likely to continue in future increasing tree mortality and thus affecting community composition of forest related organisms [8,9]. Mediterranean forests, which have suffered from degradation and exploitative land use for millennia, have to adapt to decreasing annual precipitation and higher temperatures limiting water availability projected for the next decades [10]. Interactive pressures from both, urbanisation and climatic changes, affected ecosystems since 4,000 years in the eastern Mediterranean which shifted e.g. from dense forests to degraded steppe habitats [11].

Anthropogenic and climatic pressures on forests will lead to changes in habitat availability and microclimatic conditions potentially affecting species richness patterns in forests. Possible directions of such changes may be derived from a macroecological perspective where species richness patterns often correlate with available energy and water-energy balance (species-energy-hypothesis) [12,13,14,15,16]. Energy plays the largest role in temperate to arctic regions, whereas the availability of water usually constrains richness in Mediterranean to tropical climates [14]. At the local scale, species richness of different taxa is usually higher on the drier, warmer and climatically more fluctuating sites, but this effect is group-specific and higher species richness of springtails, mosses and woody plants is found on more humid and climatically more stable sites [17,18,19,20,21]. However, the relationship between species richness and temperature becomes weak and variable towards small spatial grain [22,23], limiting our understanding of underlying processes probably because of site-specific effects.

Large valleys with opposing slopes may act as model systems for strong climatic gradients where the impact of a changing climate can be studied on the same species pool without regional differences in soil type and landscape history [24]. Here we use the ‘Evolution Canyon’ model system in Israel to investigate effects of microclimatic divergence and resulting differences in forest structure on saproxylic beetles. This group of organisms represent a large proportion of forest biodiversity and play a significant role in forest ecosystem functioning [25,26,27]. It has been shown that climatic conditions determine reproductive success, larval development and distribution patterns of saproxylic beetles and there is evidence that beetles respond more rapidly to changes of local climate conditions than other organisms (birds, plants), which make them suitable for bioindication [28,29,30,31,32]. In addition to a shift of species ranges and seasonal shifts of life cycle, a reduced body size is considered as the third universal ecological response to climate warming in aquatic systems, but may be also found in terrestrial habitats [33].

The aim of this study was I) to investigate the structure, richness, diversity, and similarity of saproxylic beetle assemblages in opposing microclimatic situations, II) to investigate slope effects on body size distribution in the beetle assemblage, and III) to identify specific beetle species which prefer conditions on either slope.

We tested the following specific hypotheses: 1) Species richness is higher on the climatically warmer and drier south-facing slope (species-energy hypothesis), 2) The physiological stress of trees on the south-facing slope lead to a higher abundance of xylophagous (= wood-boring) beetles (plant stress hypothesis) [34], 3) Mean body size of the beetle assemblage decreases with increasing temperature due to a shift in the proportion of small size species (community body size shift hypothesis–species shift hypothesis) [33].

This study is part of a multi-taxa research program to highlight the determinants of biodiversity distribution from cyanobacteria to mammals [17,20,24,35,36,37].

## Materials and Methods

### Ethics statement

The land where we made our field study is a public one and it is part of the Mt. Carmel National Park. The permit No. 2011/38129 was provided by the Israel Nature and National Parks Authority. No protected species were collected.

### Study area

The ‘Evolution Canyon’ (= EC) is located at lower Nahal Oren, Mt. Carmel, Israel (32°43’N, 34°58’E). The EC runs in an east-west direction and thus consists of a south-facing (SFS) and a north-facing slope (NFS). Interslope distance is 400 m at the top of the valley and 100 m at the bottom. A savanna-like open park forest vegetation of evergreen *Ceratonia siliqua* and *Pistacia lentiscus* and tall grasses covers the xeric SFS, while a dense Mediterranean forest of evergreen *Quercus calliprinos*, *Ceratonia siliqua*, and deciduous *Pistacia palaestina* covers the NFS. The described vegetation patterns are a result of the slope aspect with a higher solar radiation on the south-facing slope leading to different microclimatic conditions between slopes [38]. Several resource-based variables important for saproxylic beetles such as canopy openness and tree density are therefore highly determined by slope aspect. We believe that in our model system it is not possible to investigate effects of canopy openness or tree density independent from slope aspect without manipulation of vegetation structure. The slopes are grazed by cows during spring in order to decrease the amount of dry biomass and thus decrease the risk of fires during summer. Perhaps, grazing could increase the proportion of the spiny plants and bushes (*Calycotome villosa*, *Sarcopoterium spinosum* etc.). The past fires could have some influence on plant composition as well. But the studied area was not influenced by fires at least during the last 20 years. No cutting of trees has been observed during the last 20 years. However, one cannot exclude the possibility that some trees were cut on the north-facing slope during 19th century when there was a big demand for charcoal. Regional macroclimatic conditions are characterized by a Mediterranean climate with 600 mm mean annual rainfall and 20.7°C mean annual temperature (mean temperatures of 13°C in January and 28°C in August). There are higher temperatures, more solar radiation, and lower relative humidity accompanied by larger microclimate fluctuations on the SFS compared with the NFS [24,39]. Annual mean temperature is 1 to 3°C higher on the SFS than on the NFS depending on elevation.

We didn’t measure dead wood volume and quality on study trees or on the study plots. Dead wood is generally scarce on both slopes due to the historic management by grazing and wood-cutting. Only small dead branches attached to the trees are found, whereas large pieces of dead wood are missing on all elevation levels studied.

### Beetle sampling, plot design, and species data

Beetles were sampled by means of flight-interception traps from March until September 2009 [40]. Catches made with those traps represent activity and population size of saproxylic beetles, but also reflect placement of the traps and thus surrounding conditions [41]. We used traps with crossed panels of plexiglass (50 x 30 cm) and a solution of ethylene glycol as a preservation agent. We focussed our study on *Ceratonia siliqua* trees which grow on both slopes and reach up to 10 m height at EC. Trees on both slopes are relatively small in diameter at breast height (DBH up to 30 cm) compared to some old individuals at valley bottom. Fifteen vital *Ceratonia siliqua* trees with similar DBH were selected at three elevation levels on each slope (five trees at each level). The lowest elevation level was at 40 to 55 m a.s.l., the mid elevation level was at 55 to 75 m a.s.l., and the upper level was at 90–120 m. Traps were placed in the lower canopy of the trees at a height of 2–4 m above ground and emptied regularly at three-weekly intervals. Distance between traps at one elevation level was ranging from 20 to 70 m due to the availability of suitable study trees.

We sorted the beetle material into families and morphospecies (hereafter named species), which is a suitable approach to examine species richness patterns and community composition in situations where identification of species is difficult [42]. In total we caught 9859 beetle individuals between March and September 2009; these belonged to 298 species representing 47 beetle families. 108 beetle species (among them 55 saproxylics) of 14 beetle families were identified to species level by taxonomic experts or by the authors (see species list in S1 Table). Further analysis of the data was made for a selection of families in which large proportions of described species show a saproxylic life-cycle, i.e. species living in wood of healthy trees were also considered [43,44,45]: Aderidae, Anobiidae, Anthribidae, Bostrychidae, Bothrideridae, Buprestidae, Cerambycidae, Cleridae, Colydiidae, Cucujidae, Curculionidae: Scolytinae, Elateridae, Endomychidae, Histeridae, Laemophloeidae, Lyctidae, Malachiidae, Melyridae, Monotomidae, Mordellidae, Mycetophagidae, Ptinidae, Scarabaeidae, Scraptiidae, Tenebrionidae. Non-saproxylic species from otherwise saproxylic families were excluded from this selection (Tenebrionidae: *Catomus lepidus*, *Gonocephalum costatum rugulosum*, *Opatroides curtulus*, *Tentyria herculeana*, Scarabaeidae: *Oxythyrea noemi*, *Sisyphus schaefferi*).

We obtained body sizes of the species by measuring the distance between the tip of mandibles/pronotum and the tip of the elytra with a Leica M80. Mean body size was calculated for each trap based on the saproxylic species it contained. The body size of saproxylic beetles in the dataset ranged from 1.4 up to 34 mm.

### Analyses

Comparison of species richness between slopes was done using individual based rarefaction curves with 95% confidence intervals [46]. Completeness of the species inventory per slope was evaluated as the percentage of observed species in relation to the number of species predicted by the abundance-based richness estimator CHAO1 with 95% confidence intervals. Calculations were done with EstimateS version 9.1.0 with 100 randomisations [47]. We applied two-way analysis of variance (ANOVA) to test for differences in the abundance, number of species and families, and species diversity (Simpson’s D) between slopes and between elevation levels using data from individual traps.

Four wood-boring families or subfamilies (Anobiidae, Bostrychidae, Cerambycidae, Curculionidae: Scolytinae) were tested for differences in abundance between slopes (= possible effects of the plant stress hypothesis).

Mean body size for each trap was calculated based on presence-absence or on abundance of the species. The association of each species for the SFS was measured by correlation analysis, and correlation coefficients were related to body size. Correlations of body size with SFS association as predicted by the community body size shift hypothesis were calculated for presence-absence and log(abundance+1) of the species.

Non-metric multidimensional scaling (NMDS) was applied to investigate assemblage similarity between traps at species level. We used only species with more than 10 individuals in the whole sample to calculate the NMDS (Bray-Curtis dissimilarity). Effects of slope aspect and elevation level on assemblage structure were tested with a PERMANOVA (999 permutations) implemented in the ‘adonis’-function.

We tested the indicator value of all 55 identified saproxylic species for both NFS and SFS with the ‘indval’-function (1000 iterations) in the labdsv-package which combines a species relative abundance with its relative frequency of occurrence [48]. Fisher’s exact test was used to compare frequency of occurrence between slopes. We carried out the statistical analyses with R 2.13.2 [49] using the libraries ‘vegan’ [50], ‘MASS’ [51], ‘ellipse’ [52]and ‘labdsv’ [53].

## Results

**Fig 1:**
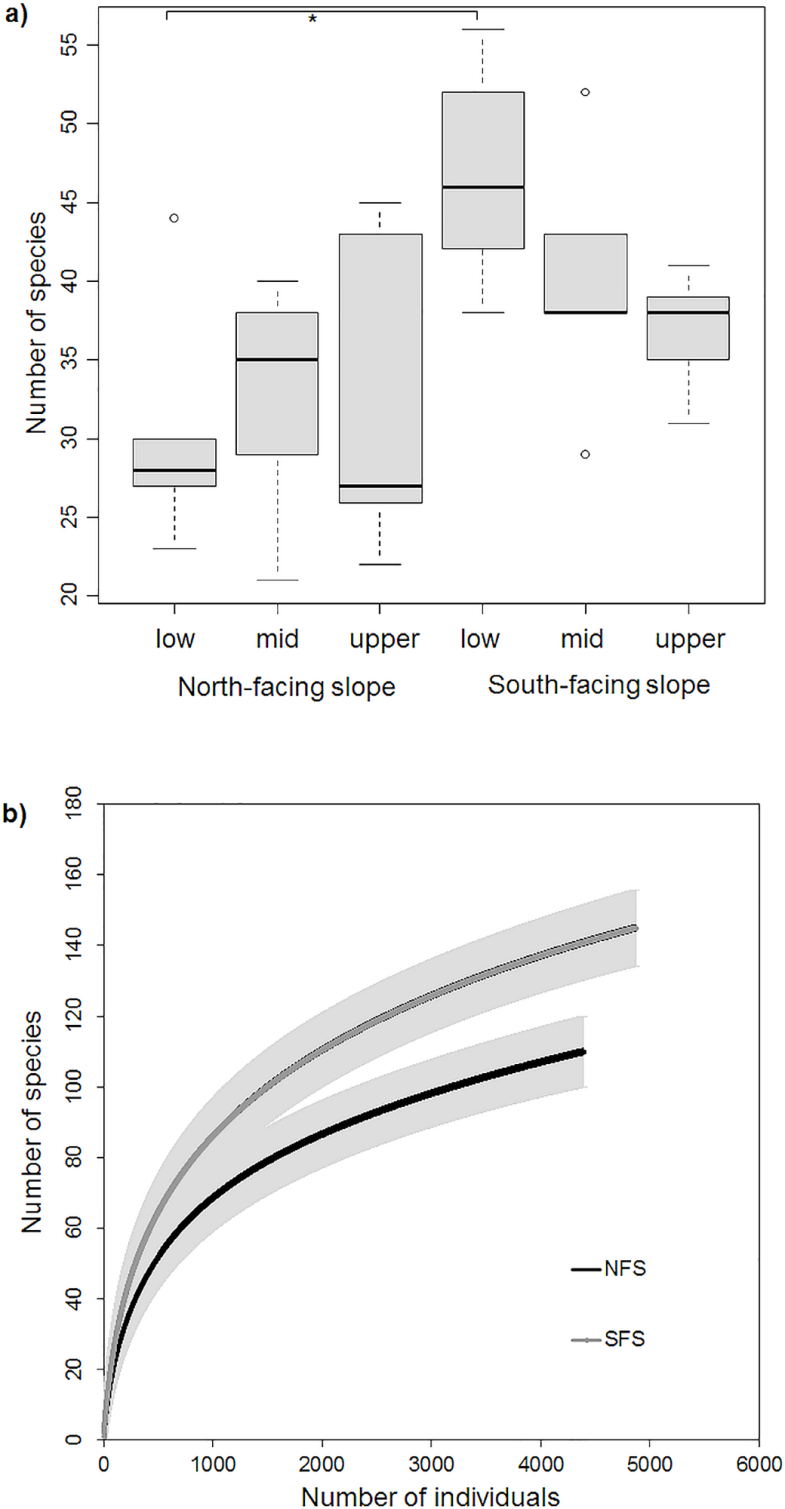
Number of saproxylic species per trap on two contrasting slopes and at three elevation levels (a); and individual-based rarefaction curves with 95% confidence intervals for the estimated richness on both slopes (b) in lower Nahal Oren, Israel. Boxplots are based on five traps per elevation level per slope. Pairwise comparisons revealed significant differences in number of species only between the two lowest elevations of the slope (see Table 1 for ANOVA, Tukey-HSD-test, p = 0.033). * p < 0.05.

We caught 9265 beetle individuals representing 169 species in 25 typical saproxylic families. Among them we found 60 species (42%) exclusively on the SFS and 25 species (21%) exclusively on the NFS. Traps contained significantly more saproxylic species on the SFS (median_SFS_ = 39 per trap) than on the NFS (median_NFS_ = 29), while elevation level had no significant effect (Fig 1A, Table 1). A similar pattern was found for the number of saproxylic beetle families. No difference in abundance or species diversity (Simpson’s D) was found between slopes or between elevation levels (Table 1). The species richness estimator (CHAO1) showed percentages of sampling completeness with 78% for the NFS (CHAO1_mean_ = 140.99, upper CI_95%_ = 184.3, lower CI_95%_ = 122.9) and respectively 80% for the SFS (CHAO1_mean_ = 180.45, upper CI_95%_ = 222.7, lower CI_95%_ = 161.2) at the species level. Species rarefaction curves showed higher species richness for the SFS, but similar abundance for both slopes (Fig 1B).

**Table 1:** Effects of slope aspect and elevation level on number of saproxylic beetle families, species, and individuals as well as diversity in the Evolution Canyon, Israel.

		Number of saproxylic beetle families		Number of saproxylic beetle species		Simpson’s D diversity		Number of saproxylic individuals	
**ANOVA**	df	*F*	*P*	*F*	*P*	*F*	*P*	*F*	*P*
**Slope**	1	4.68	**0.041**	10.46	**0.003**	0.55	0.467	0.84	0.370
**Elevation**	2	0.50	0.611	0.62	0.549	0.80	0.461	1.87	0.176
Slope*elevation	2	2.94	0.072	1.60	0.222	0.52	0.603	2.46	0.106
**Error**	24								

Abbreviations: df = degrees of freedom and F = F-value. Significant effects are shown in bold.

**Fig 2:**
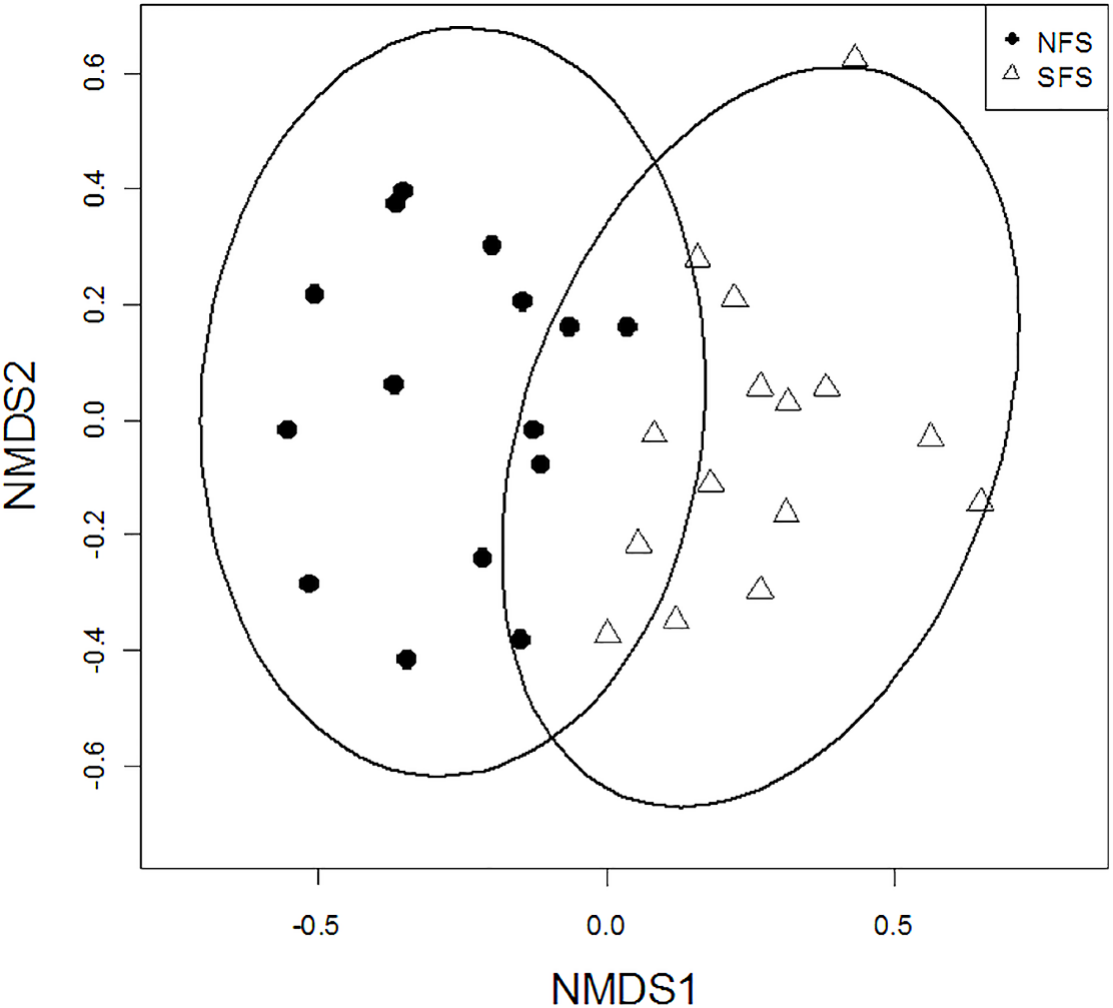
Non-metric multidimensional scaling of beetle assemblages (species level) in lower Nahal Oren on two dimensions using Bray-Curtis distance between traps. Symbols represent traps on the north-facing slope (NFS) and south-facing slope (SFS). Stress = 0.165, PERMANOVA slope effect p = 0.001, effect of elevation level p = 0.093. Ellipsoids represent 95% confidence regions of the sites of a given slope.

Of the four wood-boring taxonomic groups tested for differences in abundance between slopes, only Anobiidae (Wilcoxon-test, p < 0.001) and Scolytinae (Wilcoxon-test, p < 0.001) showed higher abundance on the SFS (Wilcoxon-test, p < 0.001), while the other two families showed similar abundance on the SFS and NFS (Bostrychidae p = 0.724, Cerambycidae p = 0.631). NMDS showed that species composition is determined by slope aspect (Fig 2). Assemblages on the NFS were significantly different from those living on the SFS, but showed little overlap in 95%—confidence regions (NMDS, k = 2, stress = 0.165, PERMANOVA slope effect: p = 0.001).

**Fig 3:**
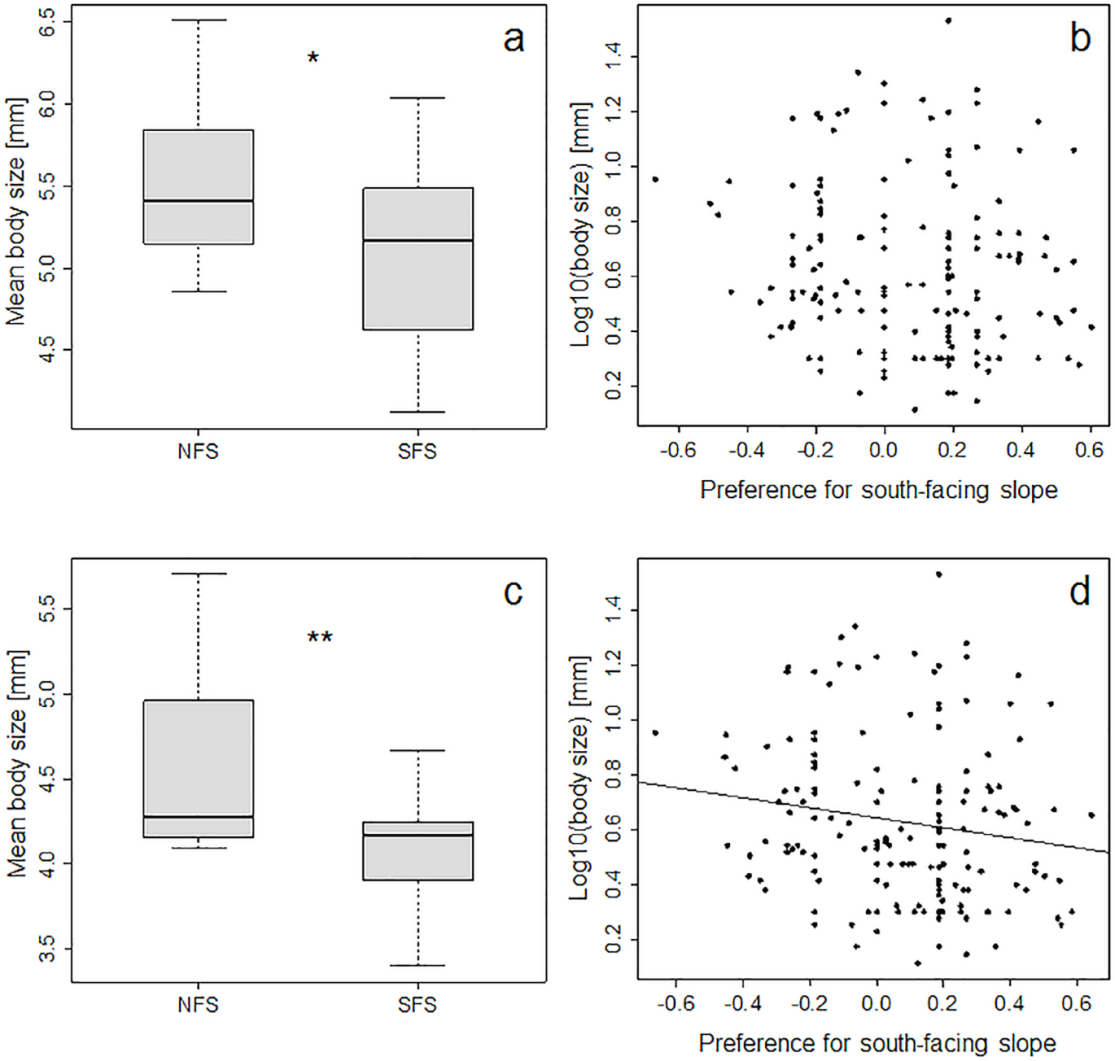
Body size of saproxylic beetles on a south-facing slope (SFS) and a north-facing slope (NFS). Boxplots of mean body size are based on 15 traps per slope. Left-hand panels show median body size per trap based on presence-absence (a), and on abundance (c). The right-hand panels show the relationship between body size and affinity to SFS of each species based on presence-absence (b) and abundance (d). Body size decreased significantly with the preference for the south-facing slope only if abundances were considered (d) (ANOVA, F_1,161_ = 4.09, p = 0.045). * p < 0.05, ** p < 0.01.

**Fig 4:**
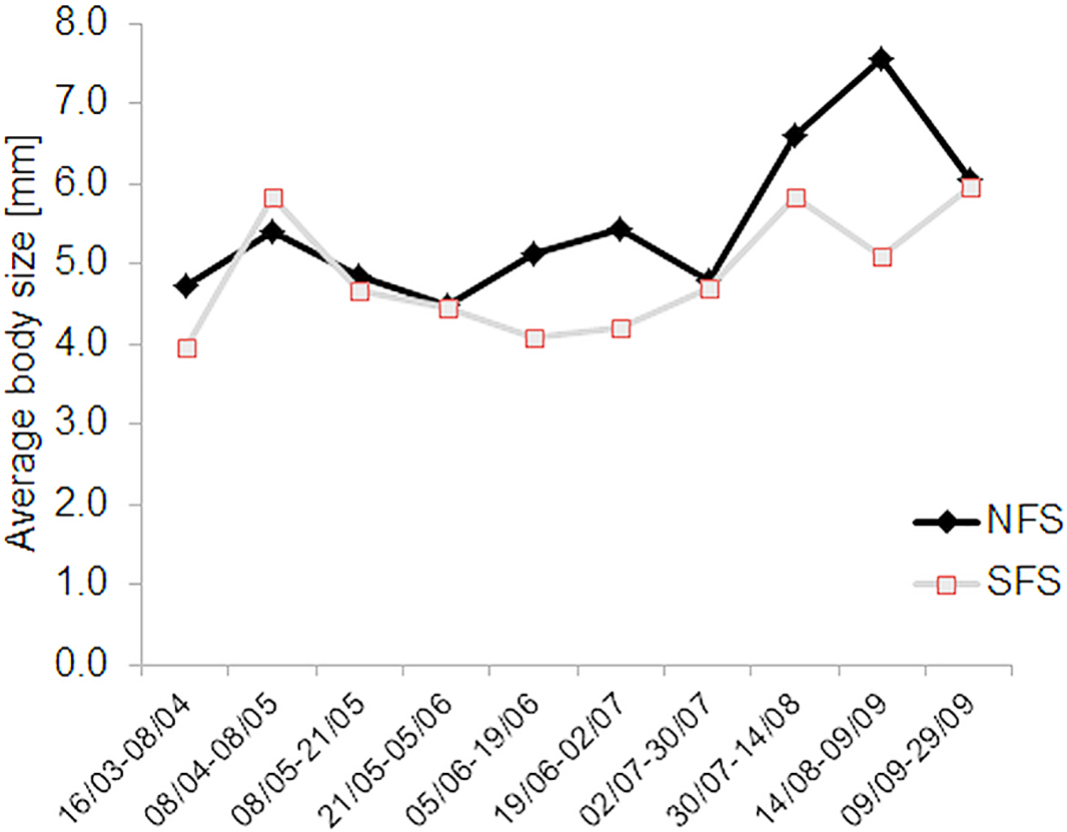
Mean average body size per sampling date on a south-facing slope (SFS) and a north-facing slope (NFS). Mean value of average body size per sampling date is based on 15 traps per slope. Calculation is based on presence-absence of species.

Body size distribution of saproxylic species was similar between the two contrasting slopes, but with a higher number of small species (< 5 mm) occurring on the SFS (ANOVA, F_1,28_ = 15.6, p < 0.001). Mean body size per trap was smaller on the SFS than on the NFS when based on presence-absence (ANOVA, F_1,28_ = 4.889, p = 0.035, Fig 3A) or abundance data (F_1,28_ = 9.697, p = 0.004, Fig 3C). Body size of saproxylic beetle species declined significantly with affinity to the SFS when affinity was calculated based on abundance (Fig 3D), but not when it was based on presence-absence (Fig 3B). Species occurring exclusively on either slope did not differ in mean body size (mean = 5.84 mm on SFS vs. mean = 5.56 mm on NFS) (Wilcoxon-test, n_NFS_ = 25, n_SFS_ = 53, p = 0.480). But we observed a lower mean body size in the beetle assemblage for the SFS during the hot and dry summer period ranging from beginning of June to mid of August (mean absolute differences in body size Δ = 0.41 to 1.62 mm, three sampling intervals, Fig 4), while this was not found for the rest of the sampling period including end of August and beginning of September (Δ = 0.05 to 0.47 mm, six sampling intervals). Lower mean body size on the SFS was also found in mid and end of September (Δ1.15 mm, Fig 4).

We tested the indicator value of all 55 identified saproxylic species and found that nine species (16%) could be used as an indicator of the environmental conditions either on the SFS (six species) or on the NFS (three species). The indicator value ranged between 0.33 and 0.72 (Table 2). Most indicator species showed a significant association with one slope, but only two species occurred exclusively on one slope. Six indicator species, including those ranked top, are only known from the east-Mediterranean region (Egypt, Israel, Jordan, Lebanon, Syria, Turkey).

**Table 2:** Beetle species with an association with a slope aspect.

Family	Species	Traps SFS	Traps NFS	Fisher’s exact test (p)	Indicator value	Distribution
**Species preferring NFS**						
Tenebrionidae	*Allecula oronthea* Baudi di Selve, 1881	8	14	0.035*	0.63*	IL, LE, TR
Tenebrionidae	*Mycetocharina syriaca* Baudi di Selve, 1881	1	8	0.014*	0.50**	SY, IL
Elateridae	*Cardiophorus sacratoides* Platia, 2010	2	9	0.021*	0.46*	IL
**Species preferring SFS**						
Curculionidae	*Xyleborus saxesenii* (Ratzeburg, 1837)	12	4	0.009**	0.72**	World-wide
Cleridae	*Phloiocopus andresi* Schenkling, 1912	10	1	0.002**	0.63**	SY, EG, IL
Bostrychidae	*Xylopertha reflexicauda* (Lesne, 1937)	12	5	0.025*	0.51*	IR, PA, IL
Bothrideridae	*Ogmoderes angusticollis* (Brisout de Barneville, 1861)	7	2	0.109	0.43*	Mediterranean
Elateridae	*Melanotus busei* Platia, 2010	5	0	0.042*	0.33*	IL
Curculionidae	*Carphoborus perrisi* (Chapuis, 1869)	5	0	0.042*	0.33*	Mediterranean, Irano-Turanian

The frequency of occurrence on the south-facing (SFS) and north-facing slope (NFS) and indicator values based on species relative frequency and relative abundance are shown (see Material and Methods for further explanation). The potential maximum number of traps occupied was 15 per slope.

*** p < 0.001,

** p < 0.01,

* p < 0.05.

## Discussion

### Species richness

We found higher species richness on the more open SFS representing warmer and drier conditions than on the NFS. This pattern might be explained by differences in microclimate (species-energy hypothesis) and by the different vegetation structure (canopy openness) between slopes.

Climate conditions play an important role in determining species richness gradients in terrestrial habitats at large spatial scales, but become weaker at small spatial extent or grain [16]. Still, we found a significant increase in species richness with temperature at very small spatial grain, which resembles patterns of other insects at larger scales. For example, species richness and beta-diversity of longhorn beetles in Europe increases with temperature [13], which probably applies to the majority of saproxylic beetles at the macroscale and locally [54,55]. Other groups of invertebrates show no or opposite relationships of species richness with temperature [56]. Species richness of butterflies within Egypt decreased strongly with temperature and to a lesser extent with precipitation [57]. Thus, saproxylic beetles are a group that responds relatively strongly and positively to higher temperatures.

However, microclimatic divergence between slopes has led to contrasting vegetation structures with only scattered trees on the SFS and a dense forest on the NFS [38]. Canopy openness and tree density are therefore highly determined by slope aspect and respective microclimatic conditions, and may further influence saproxylic beetles. While tree density per se is unlikely to affect beetle species richness [58], the more sun-exposed wood in open stands may contribute to the increased species richness on the SFS [59,60,61,62]. Irrespective of a possible proximate role of vegetation structure, the observed increase in saproxylic beetle richness with temperature fits to large-scale spatial patterns of species richness distribution and to patterns observed at a topoclimatic level in Central Europe [16,55].

### Physiological stress

Xylophagous (= wood-boring) beetles are expected to be more abundant and more frequent on the SFS than on the NFS according to the plant stress hypothesis [34,63,64]. Anobiidae and Scolytinae showed higher abundance on the SFS than on the NFS, but no difference was found for Cerambycidae and Bostrychidae. Frequency of occurrence in five Anobiid species and *Xyleborus saxesenii* (Curculionidae: Scolytinae) was higher on the SFS.

Trees growing in a warmer (micro-) climate are likely suffering from drought stress resulting in a lower resistance to attacks by small-bodied wood-boring beetles [65]. Previous studies in our study site showed that drier and warmer microclimatic conditions led to smaller leaves in the most frequent tree species (e.g. *Quercus calliprinos*, *Pistacia palaestina*) and also asymmetric leaves in some of them [66]. Smaller leaves indicate that microclimate is a selective pressure on trees, while leave asymmetry is considered to reflect physiological stress [67,68].

Most Anobiidae, Bostrychidae and *X*. *saxesenii* use already dry dead wood for breeding and are not involved in killing the tree [43,54]. Direct effects of weakened trees assumed in the plant stress hypothesis are unlikely for those groups. Higher abundance on the SFS suggests that more dry dead wood is available for breeding due to physiological stress of trees (indirect effects of stress). Another explanation could be that flight activity of beetles is higher on the SFS due to warmer conditions only suggesting differences in beetle density. In another study, trap samples were consistent with rearing data and thus well reflect true variation in abundance and species richness [55]. However, the density of wood-borers attacking living trees such as most Scolytinae likely increases on weakened trees stressed by drought where intensity of plant defense is lower than in healthy trees [63]. In addition, higher population densities may be directly determined by higher temperature, because fecundity and voltinism, i.e. number of generations per year, in saproxylics seem to be temperature-dependent [69,70]. Observations in wood cut from trees or woody baits exposed on both slopes are necessary to conclude direct effects of microclimatic conditions on beetle density. Evidence for the plant stress hypothesis is relatively weak in our data.

### Body size

Species traits such as body size may be affected by global warming at the individual species level and also at the community level in a similar way as in latitudinal or altitudinal gradients of temperature [71,72,73]. As warm regions tend to be inhabited by small-sized species, an increase of the proportion of small size species is expected under global warming (community body size shift hypothesis–species shift hypothesis) [33]. As expected, the studied beetle assemblage was affected by slope aspect, and higher numbers of smaller beetles (< 5 mm) were found on the SFS. In accordance with this, association with the warm and dry conditions on the SFS was negatively related to body size.

Patterns of interspecific body size variation may be explained by a range of mechanisms [71,72,74], but interspecific body size clines are much more difficult to interpret than intraspecific variation [75]. Decreases of body size with temperature may be explained by lower need for energy reserves in warm environments (starvation resistance), which is unlikely to apply for saproxylic beetles in Mediterranean climate. Accelerated maturation in combination with smaller adult body size at high temperatures is widespread in animals [76]. However, this ontogenetic effect applies mostly at the intraspecific level, and hence does not explain the pattern observed here which was due to higher dominance of small-bodied species on the SFS. Alternatively, observed differences in body size could arise from between-group differences, as large-bodied families are replaced by small-bodied families on the SFS [77]. However, there were only three beetle families with a total of nine individuals that occurred exclusively on the SFS.

We suggest that two mechanisms may underlie the interspecific decreases in body size with temperature: First, the observed body size cline may be related to different thermoregulatory properties of large versus small beetles (Shepherd et al. 2008). A lower mean body size of the assemblage on the SFS compared to the NFS was visible during hot summer sampling intervals when maximum air temperatures of at least 32°C occur in the Canyon. Small species may have an advantage under high temperatures, because of a reduced heat retention [78]. In other words, the risk of overheating may be higher for large species on the SFS. On the other hand, large species should have an advantage in cooler environments by retaining heat better than small species. While conditions on the NFS were probably not cool enough to completely limit development of small species, large species could benefit from better heat retention during temporary drops in temperature, such as during nights.

Second, metabolic theory of ecology can explain the observed smaller body size on the warmer SFS [79,80]. Based on biochemical kinetics and the energetic-equivalence rule (constancy of energy flux of populations per unit area), metabolic theory predicts decreasing individual body mass and population density, but increasing species richness for ectothermic animals towards high temperatures. This prediction accords both with the higher species richness and lower body size on the SFS compared to the NFS in our study. Thus, metabolic theory can explain the two main patterns found in our study, although additional specific studies are needed to clarify the possible role of thermoregulatory properties. We also plan to investigate intraspecific effects of slope orientation on body size of saproxylic beetles. Given the match between the observed effects of microclimate and macroecological patterns, the complementary use of micro- and macroclimatic gradients is encouraged to understand biotic changes caused by climate warming.

## Supporting Information

S1 Table List of families and identified species (Insecta: Coleoptera) caught with flight-interception traps at Nahal Oren (Evolution Canyon), Israel. NFS = north-facing slope, SFS = south-facing slope.
(DOCX)

S2 Table Trap-level data and data prepared for Estimates. (XLSX)
