# Supplementary material for: Microclimatic Divergence in a Mediterranean Canyon Affects Richness, Composition, and Body Size in Saproxylic Beetle Assemblages
Source: PLoS One. 2015 Jun 5;10(6):e0129323. doi: 10.1371/journal.pone.0129323 (PMC4457890; doi:10.1371/journal.pone.0129323)
Supplement: S1 Table — NFS = north-facing slope, SFS = south-facing slope. (DOCX) [file pone.0129323.s001.docx]

| Family | Species | Individuals on NFS | Individuals on SFS | Total individuals |
| --- | --- | --- | --- | --- |
| Aderidae | *.* | 3 | 21 | 24 |
| Anobiidae | *.* | 287 | 747 | 1034 |
| Anthicidae | *.* | 5 | 24 | 29 |
| Anthribidae | *.* | 37 | 27 | 64 |
| Apionidae | *.* | 10 | 7 | 17 |
| Bostrychidae | *Apate monachus* F., 1775 | 54 | 51 | 105 |
| Bostrychidae | *Paraxylogenes reflexicauda* (Lesne, 1937) | 15 | 27 | 42 |
| Bostrychidae | *Scobicia chevrieri* (Villa & Villa, 1835) | 1704 | 1989 | 3693 |
| Bostrychidae | *Sinoxylon ceratoniae* (L., 1758) | 1 | 1 | 2 |
| Bostrychidae | *.* | . | 3 | 3 |
| Bothrideridae | *Ogmoderes angusticollis* (Brisout de Barneville, 1861) | 2 | 21 | 23 |
| Bruchidae | *.* | 12 | 61 | 73 |
| Buprestidae | *Acmaeodera saxicola* Spinola, 1838 | 1 | . | 1 |
| Buprestidae | *Agrilus tenuissimus* Abeille de Perrin, 1891 | 1 | . | 1 |
| Buprestidae | *Agrilus cf. roscidus* Kiesenwetter, 1857 | . | 1 | 1 |
| Buprestidae | *Anthaxia laticeps navratili* Bílý, 1984 | . | 1 | 1 |
| Buprestidae | *Anthaxia olympica astoreth* Obenberger, 1937 | 7 | 4 | 11 |
| Buprestidae | *Anthaxia perrini* Obenberger, 1918 | . | 3 | 3 |
| Buprestidae | *Anthaxia sponsa* Kiesenwetter, 1857 | . | 1 | 1 |
| Buprestidae | *Anthaxia jordanensis* Bílý, 1984 | 2 | 1 | 3 |
| Buprestidae | *Capnodis cariosa* (Pallas, 1776) | . | 1 | 1 |
| Buprestidae | *Galbella felix* Marseul, 1865 | . | 2 | 2 |
| Cantharidae | *.* | 3 | 1 | 4 |
| Carabidae | *Singilis plagiatus* (Reiche & Saulcy, 1855) | 1 | 50 | 51 |
| Carabidae | *.* | 6 | 4 | 10 |
| Catopidae | *.* | 1 | . | 1 |
| Cerambycidae | *Agapanthia spec.* | . | 1 | 1 |
| Cerambycidae | *Alocerus moesiacus* (Frivaldszky, 1838) | 1 | . | 1 |
| Cerambycidae | *Axinopalpis gracilis* (Krynicki, 1832) | 1 | . | 1 |
| Cerambycidae | *Chlorophorus varius damascenus* (Chevrolat, 1854) | . | 1 | 1 |
| Cerambycidae | *Clytus rhamni* (Germar, 1817) | 1 | . | 1 |
| Cerambycidae | *Deilus fugax* (Olivier, 1790) | 3 | . | 3 |
| Cerambycidae | *Nathrius brevipennis* (Mulsant,1839) | 18 | 20 | 38 |
| Cerambycidae | *Niphona picticornis* Mulant, 1839 | 16 | 7 | 23 |
| Cerambycidae | *Penichroa fasciata* (Stephens, 1831) | 17 | 22 | 39 |
| Cerambycidae | *Deroplia genei genei* (Aragona, 1830) | 1 | . | 1 |
| Cerambycidae | *Stenopterus rufus syriacus* Pic, 1892 | . | 1 | 1 |
| Cerambycidae | *Stromatium unicolor* (Olivier, 1795) | 5 | 4 | 9 |
| Cerambycidae | *Xylotrechus stebbingi* Gahan, 1906 | 1 | 2 | 3 |
| Chrysomelidae | *.* | 4 | 25 | 29 |
| Clambidae | *.* | . | 1 | 1 |
| Cleridae | *Denops albofasciatus* (Charpentier, 1825) | 64 | 43 | 107 |
| Cleridae | *Korynetes coxalis* Reitter, 1894 | 1 | 1 | 2 |
| Cleridae | *Phloiocopus andresi* Schenkling, 1912 | 1 | 19 | 20 |
| Cleridae | *Tarsostenus univittatus (*Rossi, 1792) | . | 1 | 1 |
| Coccinellidae | *.* | 8 | 41 | 49 |
| Colydiidae | *.* | 6 | 4 | 10 |
| Corylophidae | *.* | 2 | 4 | 6 |
| Cryptophagidae | *.* | 1 | 9 | 10 |
| Cucujidae | *Oryzaephilus surinamensis* (L.,1758) | 4 | 5 | 9 |
| Cucujidae | *.* | . | 4 | 4 |
| Curculionidae | *.* | 4 | 7 | 11 |
| Curculionidae: Scolytinae | *Carphoborus perrisi* (Chapuis, 1869) | . | 9 | 9 |
| Curculionidae: Scolytinae | *Coccotrypes carpophagus* (Hornung, 1842) | 2 | 10 | 12 |
| Curculionidae: Scolytinae | *Coccotrypes dactyliperda* (Fabricius, 1801) | 5 | 5 | 10 |
| Curculionidae: Scolytinae | *Crypturgus numidicus* Ferrari, 1867 | 2 | 4 | 6 |
| Curculionidae: Scolytinae | *Hylurgus miklitzi* Wachtl, 1881 | 2 | 4 | 6 |
| Curculionidae: Scolytinae | *Orthotomicus erosus* (Wollaston, 1857) | . | 2 | 2 |
| Curculionidae: Scolytinae | *Phloeotribus scarabaeoides* (Bernard, 1788) | . | 3 | 3 |
| Curculionidae: Scolytinae | *Xyleborus saxesenii* (Ratzeburg, 1837) | 5 | 43 | 48 |
| Curculionidae: Scolytinae | *.* | 3 | 4 | 7 |
| Dermestidae | *.* | 8 | 14 | 22 |
| Elateridae | *Adelocera pygmaea* (Baudi, 1871) | . | 6 | 6 |
| Elateridae | *Cardiophorus convexithorax* Desbrochers, 1870 | . | 3 | 3 |
| Elateridae | *Cardiophorus sacratoides* Platia, 2010 | 10 | 3 | 13 |
| Elateridae | *Drasterius bimaculatus* (Rossi, 1790) | . | 1 | 1 |
| Elateridae | *Lacon candezei* (Desbrochers, 1875) | 4 | 3 | 7 |
| Elateridae | *Lacon punctatus* (Herbst, 1779) | . | 2 | 2 |
| Elateridae | *Lanelater judaicus* (Reiche & Saulcy, 1857) | . | 2 | 2 |
| Elateridae | *Melanotus busei* Platia, 2010 | . | 8 | 8 |
| Elateridae | *Melanotus fusciceps* (Gyllenhal, 1817) | 2 | 1 | 3 |
| Elateridae | *Melanotus orbachorum* (Platia, 2010) | . | 1 | 1 |
| Elateridae | *Mulsanteus guillebelli* (Mulsant & Godart, 1853) | 28 | 24 | 52 |
| Endomychidae | *.* | . | 2 | 2 |
| Histeridae | *.* | . | 2 | 2 |
| Hydrophilidae | *.* | . | 6 | 6 |
| Laemophloeidae | *.* | 2 | 9 | 11 |
| Lampyridae | *.* | 5 | 31 | 36 |
| Lathridiidae | *.* | 6 | 17 | 23 |
| Leiodidae | *.* | . | 2 | 2 |
| Lyctidae | *.* | . | 5 | 5 |
| Malachiidae | *Hypebaeus vesiculiger* Marseul, 1868 | 1 | 3 | 4 |
|  | *Callotroglops eburifer* Peyron, 1877 | 1 | 1 | 2 |
|  | *Colotes florieni* Pic, 1911 | 1 | 1 | 2 |
|  | *Malachius coccineus* Waltl, 1838 | . | 2 | 2 |
| Melyridae | *.* | 1676 | 1459 | 3135 |
| Monotomidae | *.* | . | 2 | 2 |
| Mordellidae | *.* | 17 | 18 | 35 |
| Mycetophagidae | *.* | 1 | 4 | 5 |
| Nitidulidae | *.* | 58 | 12 | 70 |
| Oedemeridae | *.* | 9 | 15 | 24 |
| Phalacridae | *.* | . | 1 | 1 |
| Ptinidae | *Dignomus aureopilis* (Desbrochers des Leges, 1875) | 73 | 43 | 116 |
| Ptinidae | *Ptinus variegatus* Rossi, 1794 | 6 | 6 | 12 |
| Ptinidae | *Ptinus peyroni* Pic, 1899 | 12 | 22 | 34 |
| Scarabaeidae | *Aplidia spec.* | 6 | 10 | 16 |
| Scarabaeidae | *Maladera syriaca* Petrovitz, 1969 | 131 | 4 | 135 |
| Scarabaeidae | *Oxythyrea noemi* Reiche & Saulcy, 1856 | . | 1 | 1 |
| Scarabaeidae | *Protaetia cuprea ignicollis* (Gory & Percheron, 1833) | 2 | 2 | 4 |
| Scarabaeidae | *Protaetia judith* (Reiche, 1871) | 5 | 2 | 7 |
| Scarabaeidae | *Sisyphus schaefferi boschniaki* Fischer, 1823 | 6 | 4 | 10 |
| Scarabaeidae | *.* | 1 | . | 1 |
| Scraptiidae | *.* | 72 | 52 | 124 |
| Scydmaenidae | *.* | . | 2 | 2 |
| Staphylinidae | *Aleochara maculipennis* Baudi di Selve, 1857 | . | 1 | 1 |
| Staphylinidae | *Aloconota gregaria* (Erichson, 1839) | . | 1 | 1 |
| Staphylinidae | *Amarochara inermis* Assing, 2002 | 1 | 2 | 3 |
| Staphylinidae | *Anotylus inustus* (Gravenhorst, 1806) | . | 1 | 1 |
| Staphylinidae | *Atheta aeneicollis* (Sharp, 1889) | 1 | . | 1 |
| Staphylinidae | *Atheta coriaria* (Kraatz, 1856) | . | 1 | 1 |
| Staphylinidae | *Atheta mucronata* (Kraatz, 1859) | . | 2 | 2 |
| Staphylinidae | *Atheta spec.* | . | 1 | 1 |
| Staphylinidae | *Bryoporus multipunctus* Hampe, 1867 | 1 | 1 | 2 |
| Staphylinidae | *Diestota guadalupensis* Pace, 1987 | 2 | . | 2 |
| Staphylinidae | *Gabronthus maritimus* (Motschulsky, 1858) | . | 2 | 2 |
| Staphylinidae | *Haploglossa villosula* (Stephens, 1832) | . | 1 | 1 |
| Staphylinidae | *Heterothops cf. minutus* Wollaston, 1860 | . | 1 | 1 |
| Staphylinidae | *Medon dilutus pythonissa* Saulcy, 1864 | 2 | 1 | 3 |
| Staphylinidae | *Medon semiobscurus* (Fauvel, 1875) | 1 | . | 1 |
| Staphylinidae | *Megalinus flavocinctus* (Hochhuth, 1849) | 3 | . | 3 |
| Staphylinidae | *Myrmecopora fugax* (Erichson, 1839) | 2 | 1 | 3 |
| Staphylinidae | *Ocypus orientis* Smetana & Davies, 2000 | 1 | . | 1 |
| Staphylinidae | *Oxytelus piceus* (L., 1767) | . | 3 | 3 |
| Staphylinidae | *Peltodonia bodemeyeri* (Bernhauer, 1936) | 2 | . | 2 |
| Staphylinidae | *Phacophallus pallidipennis* (Motschulsky, 1858) | . | 1 | 1 |
| Staphylinidae | *Philonthus spec.* | . | 1 | 1 |
| Staphylinidae | *Placusa adscita* Erichson, 1839 | 1 | . | 1 |
| Staphylinidae | *Platystethus cf. nitens* (Sahlberg, 1832) | . | 3 | 3 |
| Staphylinidae | *Platystethus nitens* (Sahlberg, 1832) | 1 | . | 1 |
| Staphylinidae | *Pronomaea spalacis* Assing, 2007 | . | 1 | 1 |
| Staphylinidae | *Quedius josue* Saulcy, 1864 | 1 | . | 1 |
| Staphylinidae | *Quedius spec.* | 1 | . | 1 |
| Staphylinidae | *Tachyporus caucasicus* Kolenati, 1846 | 4 | 1 | 5 |
| Staphylinidae | *Tachyporus hypnorum* (Fabricius, 1775) | 2 | . | 2 |
| Staphylinidae | *Tachyporus nitidulus* (Fabricius, 1781) | 27 | 32 | 59 |
| Staphylinidae | *Xantholinus gridellii* (Coiffait, 1956) | 2 | 3 | 5 |
| Staphylinidae | *Xantholinus rufipennis* (Erichson, 1839) | 1 | . | 1 |
| Tenebrionidae | *Allecula oronthea* Baudi di Selve, 1881 | 43 | 21 | 64 |
| Tenebrionidae | *Catomus lepidus* Reitter, 1922 | . | 2 | 2 |
| Tenebrionidae | *Gonocephalum costatum rugulosum* Küster, 1849 | . | 1 | 1 |
| Tenebrionidae | *Hymenalia graeca* Seidlitz, 1896 | . | 1 | 1 |
| Tenebrionidae | *Lyphia tetraphylla* Fairmaire, 1859 | . | 1 | 1 |
| Tenebrionidae | *Mesomorphus longulus* (Reiche & Saulcy, 1857) | . | 2 | 2 |
| Tenebrionidae | *Mycetochara ruficollis* Baudi di Selve, 1881 | 7 | . | 7 |
| Tenebrionidae | *Mycetocharina syriaca* Baudi di Selve, 1881 | 14 | 1 | 15 |
| Tenebrionidae | *Opatroides curtulus* Fairmaire, 1892 | . | 1 | 1 |
| Tenebrionidae | *Prionychus cisteloides* | . | 2 | 2 |
| Tenebrionidae | *Strongylium saracenum* (Reiche & Saulcy, 1857) | 2 | . | 2 |
| Tenebrionidae | *Tentyria herculeana* Reiche & Saulcy, 1857 | . | 6 | 6 |
| Tenebrionidae | *.* | . | 2 | 2 |
| Total | *.* | 4595 | 5264 | 9859 |
